# Microfibril-associated glycoprotein 4 (Mfap4) regulates haematopoiesis in zebrafish

**DOI:** 10.1038/s41598-020-68792-8

**Published:** 2020-07-16

**Authors:** Sheena L. M. Ong, Ivo J. H. M. de Vos, M. Meroshini, Yogavalli Poobalan, N. Ray Dunn

**Affiliations:** 1 Institute of Medical Biology, Agency for Science, Technology and Research (A*STAR), 8A Biomedical Grove, #06-06 Immunos, Singapore, 138648 Singapore; 2 Skin Research Institute of Singapore, Agency for Science, Technology and Research (A*STAR), 11 Mandalay Road, Clinical Sciences Building, #17-01, Singapore, 308232 Singapore; 3 Lee Kong Chian School of Medicine, Nanyang Technological University, Clinical Sciences Building, 11 Mandalay Road, Clinical Sciences Building, Singapore, 308232 Singapore; 4 Present Address: Department of Pathology, Leiden University Medical Center, 2300 RC Leiden, The Netherlands; 5 Present Address: Department of Genetics, University Medical Center Groningen, 9700 RB Groningen, The Netherlands; 6 Present Address: Engine Biosciences, 160 Robinson Road, 23-20 SBF Center, Singapore, 068914 Singapore

**Keywords:** Haematopoiesis, Erythropoiesis, Myelopoiesis, Developmental biology

## Abstract

Microfibril-associated glycoprotein 4 (MFAP4) is an extracellular matrix protein belonging to the fibrinogen-related protein superfamily. MFAP4 is produced by vascular smooth muscle cells and is highly enriched in the blood vessels of the heart and lung, where it is thought to contribute to the structure and function of elastic fibers. Genetic studies in humans have implicated *MFAP4* in the pathogenesis of Smith-Magenis syndrome, in which patients present with multiple congenital abnormalities and mental retardation, as well as in the severe cardiac malformation left-sided congenital heart disease. Comprehensive genetic analysis of the role of MFAP4 orthologues in model organisms during development and tissue homeostasis is however lacking. Here, we demonstrate that zebrafish *mfap4* transcripts are detected embryonically, resolving to the macrophage lineage by 24 h post fertilization. *mfap4* null mutant zebrafish are unexpectedly viable and fertile, without ostensible phenotypes. However, tail fin amputation assays reveal that *mfap4* mutants have reduced numbers of macrophages, with a concomitant increase in neutrophilic granulocytes, although recruitment of both cell types to the site of injury was unaffected. Molecular analyses suggest that loss of Mfap4 alters the balance between myeloid and lymphoid lineages during both primitive and definitive haematopoiesis, which could significantly impact the downstream function of the immune system.

## Introduction

*MFAP4* (*Microfibril-associated glycoprotein 4*) encodes an extracellular matrix (ECM) protein that belongs to the fibrinogen-related protein superfamily. *MFAP4* was first associated with Smith-Magenis syndrome (SMS, OMIM #182290), a rare sporadic disorder characterized by skeletal anomalies and intellectual deficit, which is predominantly caused by a 3.7 megabase (Mb) interstitial 17p11.2 deletion encompassing the *MFAP4* locus^1^. Subsequently, copy number variant (CNV) analysis in 464 individuals identified *MFAP4* as a candidate gene for the severe cardiac malformations associated with left-sided congenital heart disease (LS-CHD)^2^. More recently, numerous studies have implicated MFAP4 as a prognostic marker (or biomarker) for assorted cancers, including ovarian^3,4^, mammary^5^ and prostate^6^, as well as hepatic and pulmonary fibrosis^7^ and chronic obstructive pulmonary disease (COPD)^8^. Despite the growing list of diseases that implicate MFAP4 in their aetiology, the exact function of MFAP4 in vivo remains largely unknown.

Biochemically, MFAP4 homo-oligomerizes into trimers and hexamers, and interacts with tropoelastin and fibrillins during elastic fiber assembly^9^ via its highly conserved fibrinogen-related (FReD) domain^10^. Immunohistochemistry revealed that MFAP4 is highly enriched in vascular smooth muscle cells (VSMCs) in the blood vessel wall of many human tissues including heart, lung, kidney, liver, testis and dermis^11,12^. In addition to elastic fiber assembly, MFAP4 is thought to play a role in cell adhesion and/or intercellular interactions through its amino (N-) terminal Arg-Gly-Asp (RGD) cell-binding motif^10,13^. Indeed, MFAP4 binds integrin α_v_β_3_ in vitro and promotes the migration and proliferation of VSMCs^14^.

We previously exploited the directed differentiation of human embryonic stem cells (hESCs) into the definitive endoderm (DE) lineage as a platform to identify novel genes upregulated after treatment with the TGFβ-related ligands Activin and BMP4^15^. *MFAP4* emerged from this screen alongside cardinal markers of the mouse primitive streak, where the mesoderm and DE emerge during gastrulation, including *T/BRACHYURY*, *CER1*, *LHX1 MIXL1* and *SOX17*. We subsequently demonstrated by chromatin immunoprecipitation coupled with massively parallel deep sequencing (ChIP-Seq) that *MFAP4* is directly bound by the Activin/Nodal effector proteins SMAD2/3, as well as the T-box transcription factor EOMESODERMIN (EOMES) in differentiating hESCs^16,17^. These findings strongly suggest that *MFAP4* is a direct target of SMAD2/3/EOMES transcriptional regulation during the earliest events of germ layer specification in the developing mammalian embryo. Given these results, we further reasoned that loss (or diminished levels) of MFAP4 during early human development could contribute to the pathologies of SMS and LS-CHD.

Anticipating strong evolutionary conservation among vertebrate MFAP4 orthologs, we embarked upon a disease modeling approach using zebrafish (*Danio rerio*). We first demonstrated that *mfap4* is indeed expressed throughout zebrafish embryonic development, but is entirely dispensable as adult *mfap4* null mutants generated by CRISPR/Cas9 gene editing are viable and fertile. To date, zebrafish *mfap4* has been most widely used as a macrophage marker^18–20^. Coupling our larval and adult *mfap4* loss-of-function zebrafish with tail fin injury models, we investigated the role of Mfap4 in the zebrafish innate immune system and revealed an unexpected role for Mfap4 in regulating lineage restriction during haematopoiesis.

## Results

### *mfap4* is expressed during zebrafish embryogenesis

**Figure 1:**
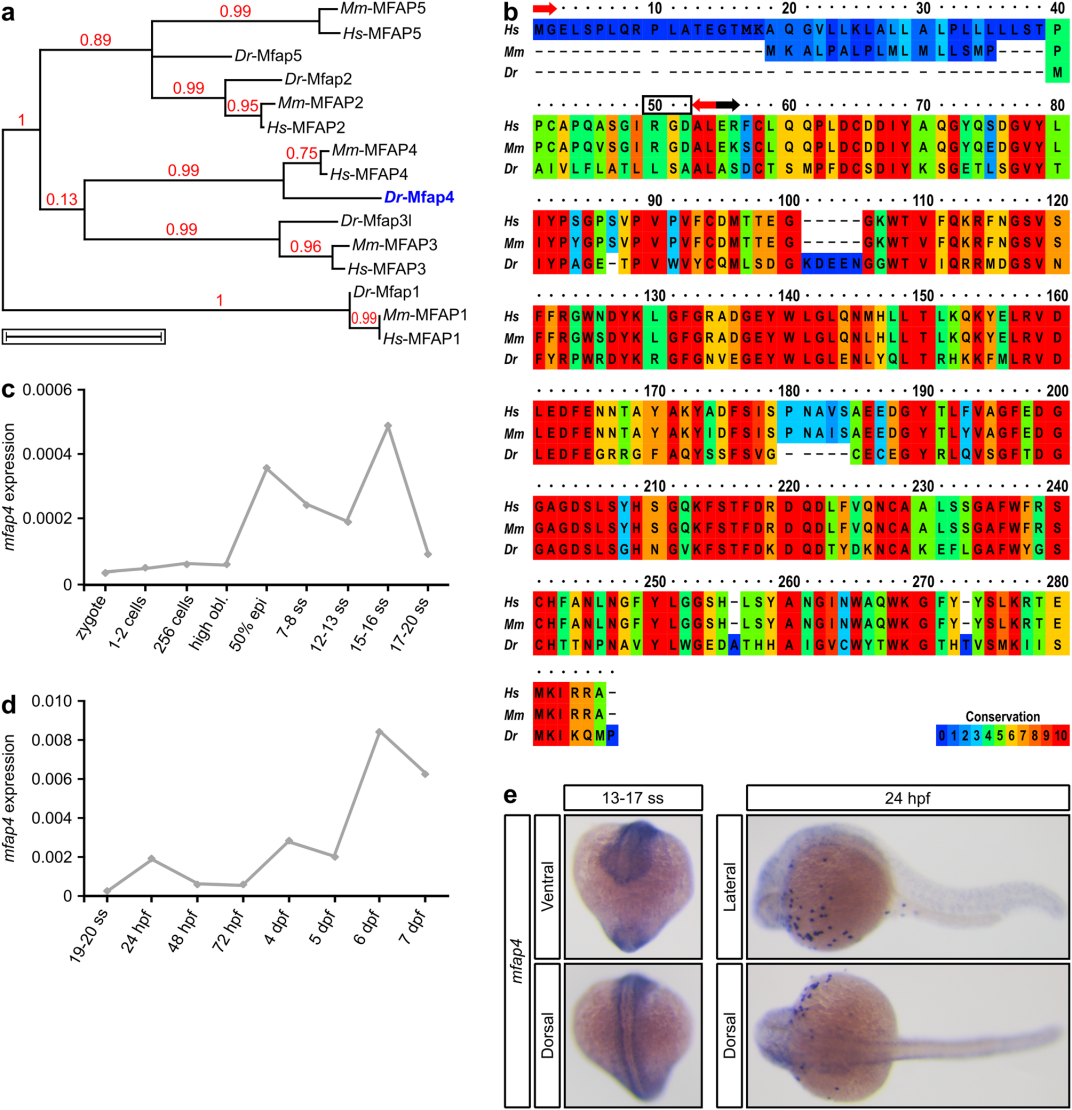
Alignment of vertebrate Mfap4 proteins and *mfap4* expression during zebrafish development. (**a**) Phylogenetic analysis of human (*Homo sapiens, Hs*) MFAP family members and their murine (*Mus musculus, Mm*) and piscine (*Danio rerio, Dr*) orthologs. Scale bar represents 1 amino acid substitution per site. (**b**) Amino acid sequence alignment of human, murine and zebrafish MFAP4 proteins displayed in ClustalX color scheme. Red arrows demarcate the start and end of the 16 amino acid signal peptide (www.uniprot.org); black box highlights the RGD sequence; black arrow indicates the start of the fibrinogen-related domain (FReD). (**c**–**e**) Zebrafish *mfap4* expression analysis by (**c**,**d**) quantitative RT-PCR (mean expression in twenty pooled embryos/larvae per time point) relative to the expression of the housekeeping gene *actin, beta 1 (actb1*) and by (**e**) whole-mount RNA in situ hybridization (purple staining)—ventral (13–17 somite stage, ss), dorsal (15–17 ss and 24 h post fertilization, hpf) and lateral (24 hpf) views. Phylogenetic analysis in panel (**a**) was performed with Phylogeny.fr online software (available at www.phylogeny.fr)^63,64^. Alignment in panel (**b**) was performed with the online PRALINE Multiple Sequence Alignment software (Vrije Universiteit Amsterdam, Centre for Integrative Bioinformatics VU, available at www.ibi.vu.nl/programs/pralinewww/). Graphs in panels (**c**) and (**d**) were generated with Microsoft Excel (for Mac 2011, version 14.7.7). This figure was created with Inkscape software version 0.92 (available at https://www.inkscape.org/).

We first established the evolutionary relationship between human, mouse and zebrafish MFAP family members. This phylogenetic analysis demonstrated that all MFAP proteins derive from a common ancestor, and that the human and mouse MFAP proteins are more closely related to each other than to their respective zebrafish orthologs (Fig. 1a). The latter is most likely due to rapid gene duplication and species divergence in response to environmental factors^10^. Interestingly, zebrafish Mfap4 is closely related to its paralog Mfap3l, as they are part of the same clade. However, there are no reports indicating any functional relationships between these two proteins.

Although many genes were duplicated during *D. rerio* evolution, zebrafish only have a single ortholog for human *MFAP4* (https://www.zfin.org)^9,21^. Protein alignment revealed that MFAP4 is well-conserved from humans to zebrafish, with 66% sequence identity and a similar domain structure, including an N-terminal signal peptide and a FReD domain^9,22^. Significantly, zebrafish Mfap4 lacks an N-terminal RGD sequence (Fig. 1b; see also^10^).

We next analyzed *mfap4* expression by quantitative RT-PCR (qPCR) and whole-mount in situ hybridization (WISH). qPCR revealed that *mfap4* is expressed in zebrafish embryos as early as the 50% epiboly stage, which is just prior to the onset of gastrulation (Fig. 1c). *mfap4* transcripts were detected throughout the segmentation period (7 to 20 somites)—a result consistent with our previous observation that *Mfap4* mRNA levels steadily increase in the developing mouse embryo during the early somite stages (Fig. 1c)^15^. From the 20 somite stage (ss), zebrafish *mfap4* levels slowly increase to around 6 days post fertilization (dpf) (Fig. 1d). These expression dynamics are supported by *mfap4* WISH of embryos at mid-segmentation stages (13–17 somites) and at 24 h post fertilization (hpf) and beyond (Fig. 1e and Supplementary Fig. S1). Increased *mfap4* expression at 24 hpf correlates with the emergence of the bloodstream (Fig. 1d)^23^. Indeed, zebrafish *mfap4* was first described by Zakrzewska et al.^19^ as a transcriptional target of Spi1b (Spi-1 proto-oncogene b), which is more commonly known as Pu.1^19^. Consistent with this finding and as previously reported, *mfap4* expression conspicuously labels macrophages (Fig. 1e and Supplementary Fig. S1).

### The generation of *mfap4*-deficient zebrafish and their phenotypic characterization

**Figure 2:**
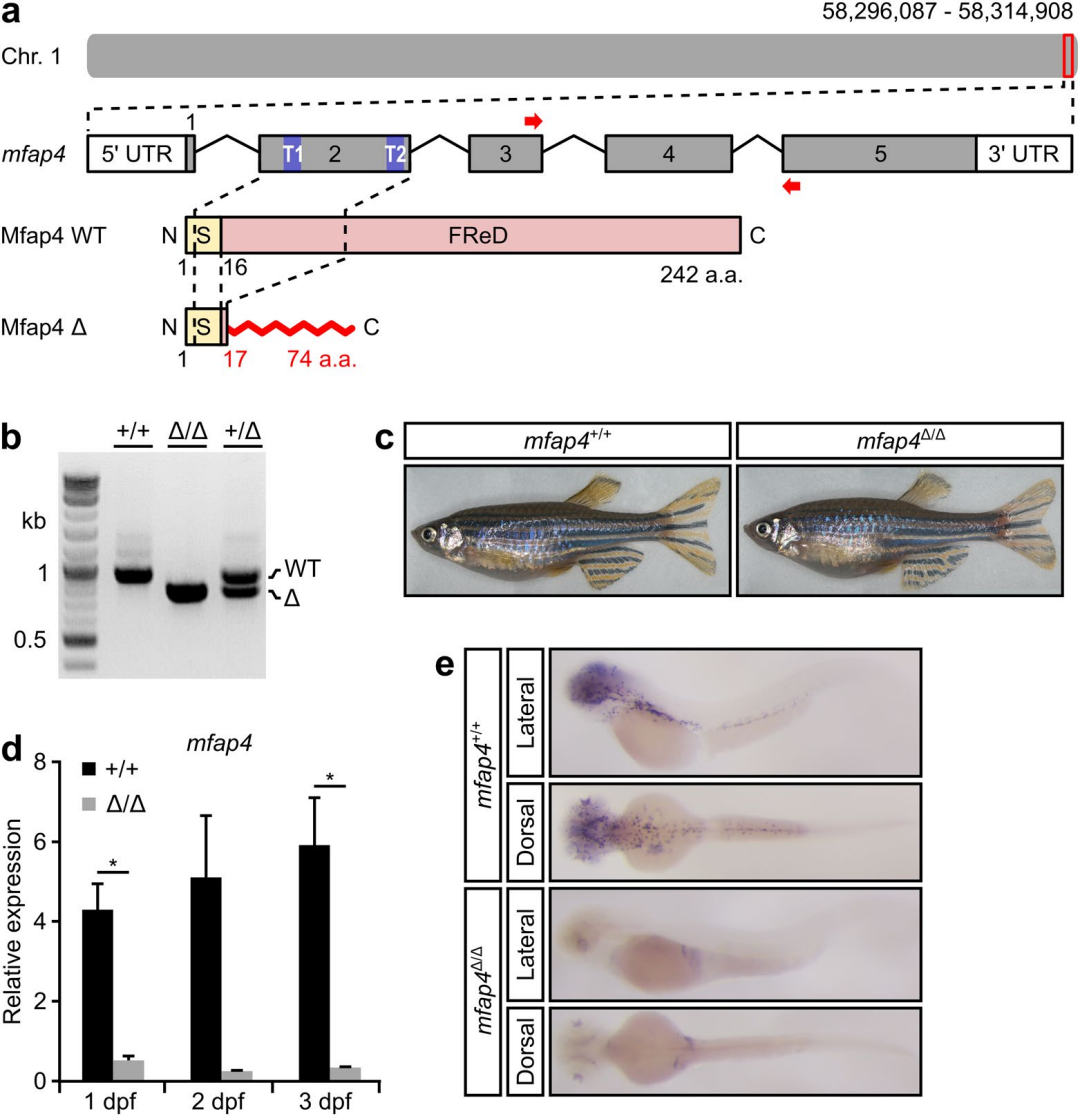
Generation of a loss-of-function mutation in zebrafish *mfap4*. (**a**) Guide RNA target sites (T1 and T2, in blue) in exon 2 for CRISPR/Cas9 gene editing of the zebrafish *mfap4* genomic locus. The resulting 150 base pair (bp) deletion is predicted to generate a truncated 74 amino acid (a.a.) protein product, p.S17Rfs58X. Abbreviations: FReD, fibrinogen-related domain; C, C-terminal; Chr, Chromosome; N, N-terminal; S, signal peptide. (**b**) Genotyping analysis of wild-type (WT, +/+) and *mfap4* heterozygous (+ /∆) and homozygous (∆/∆) mutants. The WT amplicon length is 946 bp and the mutant amplicon length is 795 bp (see Materials and Methods for additional details). The full gel image can be found in Fig. S5. (**c**) Representative images of 4-month-old WT and *mfap4*^*∆/∆*^ fish. (**d**) Quantitative RT-PCR analysis with primers indicated in panel (**a**) by red arrows relative to *actb1* reveals that the mutant *mfap4* transcript is barely detectable (significant at 1 dpf (*P* = 0.029612) and 3 dpf (*P* = 0.041617), but not at 2 dpf (*P* = 0.084486); assessed by Student’s *t*-test), which suggests the *mfap4*^*p.S17Rfs58X*^ mutant transcript is degraded. Error bars represent the standard error of the mean (s.e.m.) of technical triplicates of 20 pooled embryos per genotype per time point. (**e**) Whole-mount in situ hybridization of *mfap4* in WT and *mfap4*^*∆/∆*^ larvae at 3 days post fertilization. The graph in panel (**d**) was generated with Microsoft Excel (for Mac 2011, version 14.7.7). This figure was created with Inkscape software version 0.92 (available at https://www.inkscape.org/).

To investigate the role of Mfap4 in zebrafish development, we used CRISPR/Cas9 to generate a 150 base pair (bp) deletion in *mfap4* (hereafter designated *mfap4*^*∆*^) using two guide RNAs that target the 5′ and 3′ ends of exon 2 (Fig. 2a,b). The resulting deletion is predicted to encode a truncated protein product of 74 amino acids (p.S17Rfs58X), leaving only the first 7% of Mfap4 intact (Fig. 2a). *mfap4*^+/∆^ heterozygous fish were intercrossed and adult *mfap4*^∆/∆^ homozygous mutants were recovered that were indistinguishable from their wild-type (WT) siblings (Fig. 2a) and were moreover fertile with a normal lifespan. Very low levels of *mfap4* mRNA were detected by qPCR in WT zygotes (Fig. 1c), suggesting minimal maternal contribution. Indeed, maternal-zygotic *mfap4*^∆/∆^ mutants are also viable and fertile.

qPCR with primers downstream of the 150 bp deletion in exon 2 failed to amplify mutant *mfap4* RNA (Fig. 2d)^24^. WISH analysis on 3 dpf *mfap4*^∆/∆^ mutants further demonstrated the loss of *mfap4* mRNA (Fig. 2e). Taken together, these results indicate that the *mfap4*^∆^ mutation is a complete loss-of-function. Interestingly, we observed increased expression of *mfap2* and *mfap3l* at various larval stages in *mfap4*^∆/∆^ mutants, suggesting that Mfap2 and Mfap31 might compensate for the lack of Mfap4, perhaps by transcriptional adaptation, and thus prevent the manifestation of an overt loss-of-function phenotype (Supplementary Fig. S2)^25^.

### *mfap4* mutants have reduced numbers of macrophages and elevated production of neutrophils

**Figure 3:**
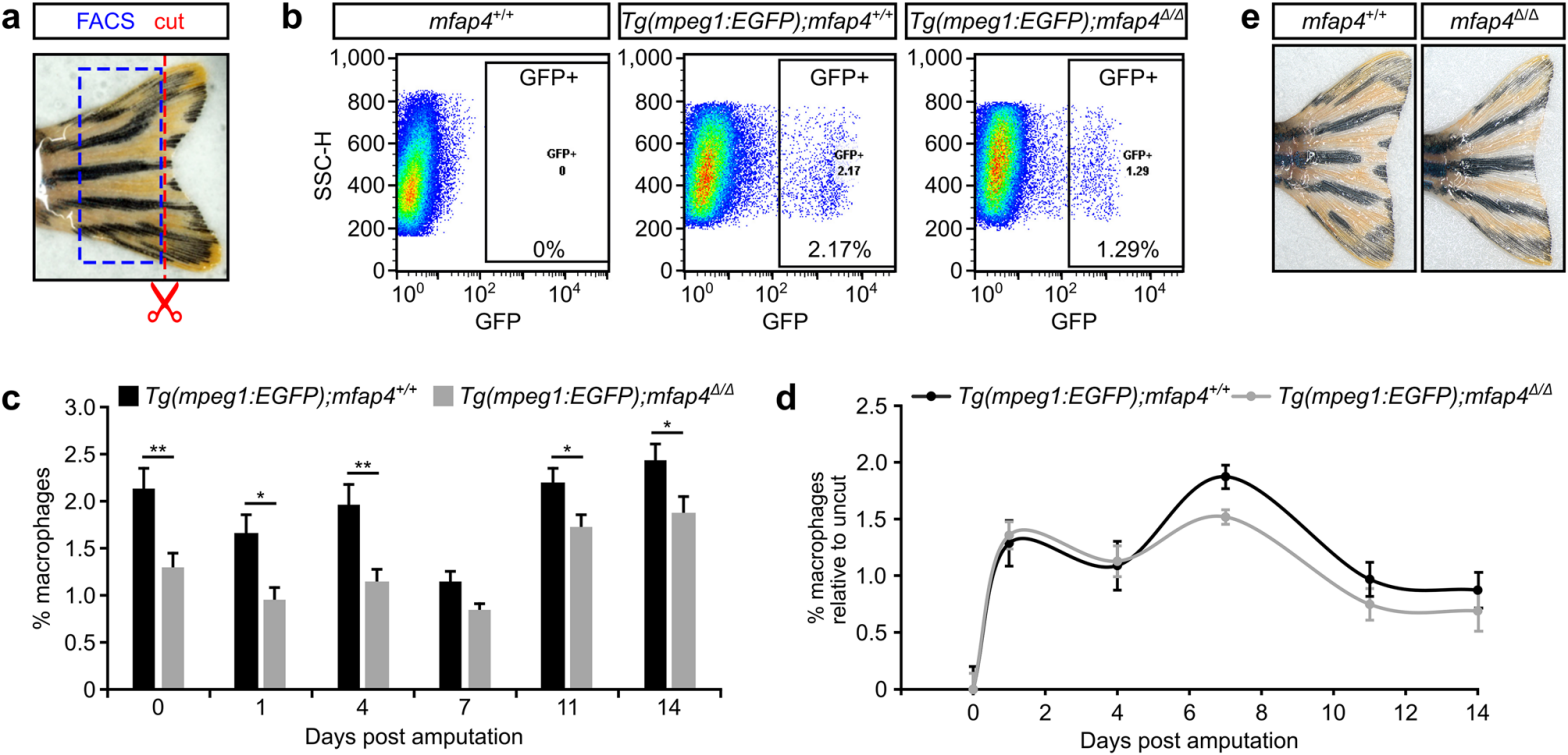
*mfap4* mutant fish have reduced numbers of macrophages. (**a**) The adult tail fins of wild-type (WT, *mfap4*^+/+^) and *mfap4*^*∆/∆*^ mutant fish were amputated (red dotted line). Cells in the remaining part of the tail fin (blue dotted box) were dissociated and macrophage numbers quantitated by FACS using the *Tg(mpeg1:EGFP)* fluorescent reporter. (**b**) FACS analysis depicting side scatter (SSC) on the *y*-axis and *Tg(mpeg1:EGFP)* fluorescence on the *x*-axis. (**c**) Graphical representation of mean macrophage numbers of fifteen biological replicates per genotype [determined as in panel (**b**)] for various time points post amputation. At most analyzed time points, there are significantly fewer macrophages in *mfap4*^*∆/∆*^ tail fins as compared to the WT fins (0 days post amputation (dpa), *P* = 0.0043; 1 dpa, *P* = 0.0264; 4 dpa, *P* = 0.0049; 7 dpa, *P* = 0.0892; 11 dpa, *P* = 0.0235; 14 dpa, *P* = 0.0145; assessed by Mann–Whitney-U test). (**d**) Quantification of macrophage numbers in tail fins at the time points shown in (**c**) relative to uncut fins. There is no delay in the recruitment of macrophages. Error bars in (**c**) and (**d**) represent the standard error of the mean (s.e.m.) of biological replicates. (**e**) Representative images of regenerated WT and *mfap4*^*∆/∆*^ tail fins two months post amputation. Images in panel (**b**) were generated with FlowJo V10 software (FlowJo LLC). Graphs in panels (**c**) and (**d**) were generated with Microsoft Excel (for Mac 2011, version 14.7.7). This figure was created with Inkscape software version 0.92 (available at https://www.inkscape.org/).

Upon tissue damage, circulating monocytes extravasate at the site of injury and differentiate into macrophages^26^. Resident macrophages then produce mediators such as cytokines to promote inflammation and elimination of apoptotic cells^26^. Recent studies have indicated a direct relationship between macrophages and tissue repair and regeneration^27,28^. Accordingly, amputated zebrafish tail fins do not fully regenerate in the absence of macrophages^28^. Given that *mfap4* expression specifically labels macrophages, we examined the response of macrophages to injury in the absence of *mfap4*. *mfap4*^∆/∆^ mutant fish were crossed into the *Tg(mpeg1:EGFP)* reporter background in which macrophages are fluorescently labelled^29^. Next, we partially amputated the tail fin primordium and assessed the effect of loss of *mfap4* on macrophage number (Supplementary Fig. S3). Flow cytometry analysis performed on 3 dpf larvae pre-amputation and immediately after amputation demonstrated considerably fewer macrophages in *mfap4*^∆/∆^ mutants as compared to WT larvae, which was statistically significant at multiple analyzed time points. However, macrophage recruitment to the amputation site was not impaired by *mfap4* loss. These findings suggest that Mfap4 plays a role in macrophage differentiation during embryonic, primitive haematopoiesis that gives rise to primitive monocytes and macrophages (see below). Around 32–36 hpf, definitive haematopoiesis commences, which gives rise to differentiated monocytes and macrophages from 5 dpf onwards^30–33^. As such, we analyzed the macrophage response in partial adult tail fin amputations (Fig. 3a,b). Similar to 3 dpf larvae, although six-month-old *mfap4*^*∆/∆*^ mutants had significantly fewer macrophages as compared to age-matched WT fish (Fig. 3c), macrophage recruitment to the amputation site was not impaired (Fig. 3d). Regenerated fins in both adult WT and *mfap4*^*∆/∆*^ mutant fish following amputation were moreover indistinguishable (Fig. 3e). These findings suggest that loss of *mfap4* quantitatively impacts the differentiation of macrophages from circulating monocytes, but those *mfap4*-deficient macrophages that do emerge show no defects in their recruitment to the site of injury.

**Figure 4:**
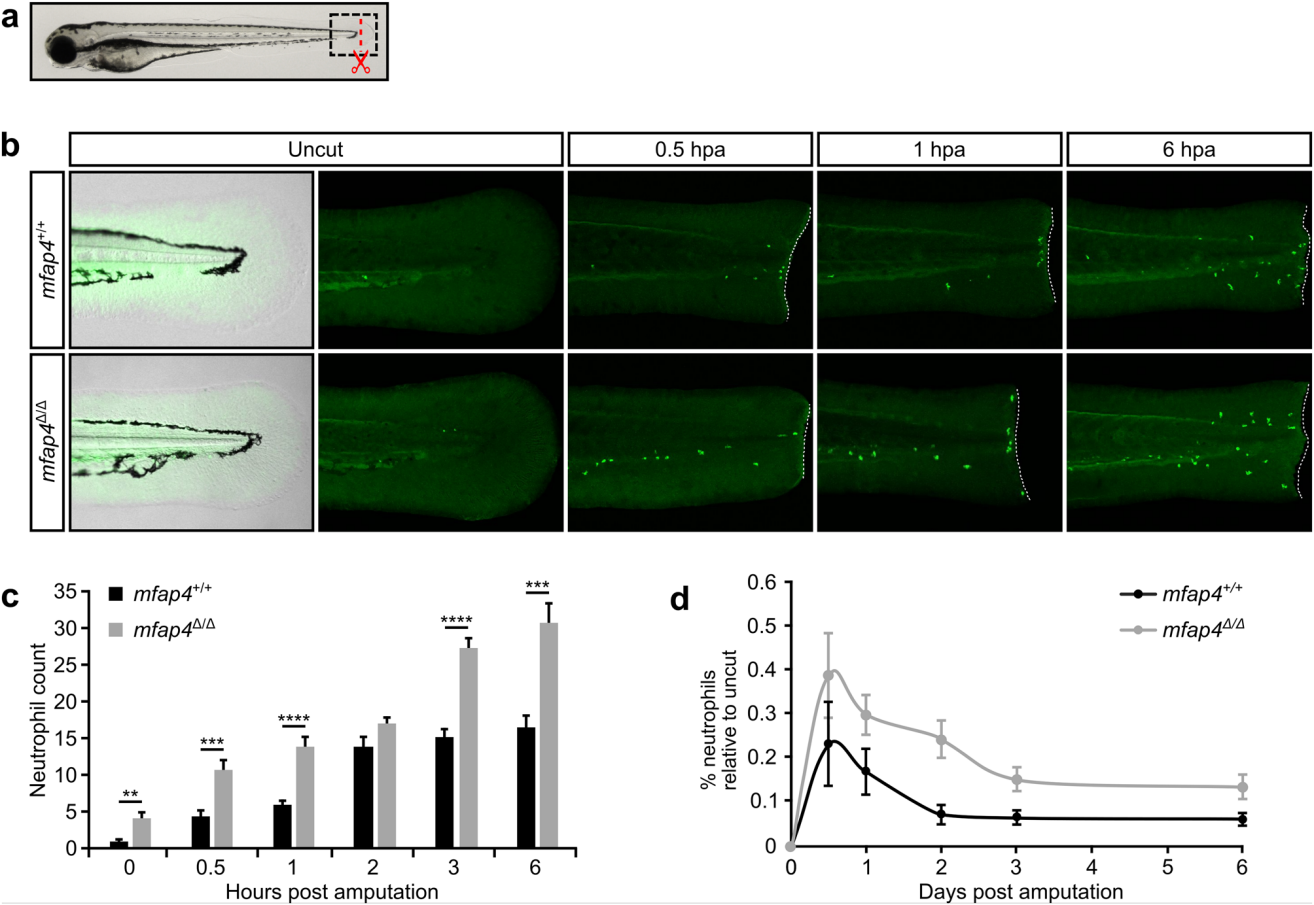
Neutrophil recruitment to the amputation site is increased in *mfap4* mutant fish. (**a**) The tail fin primordium of 3 dpf wild-type (WT, *mfap4*^+/+^) or *mfap4*^*∆/∆*^ mutant larvae was amputated (red line) and subsequently analyzed by whole-mount immunofluorescence with the neutrophil marker *mpx* (black box). (**b**) Representative images of various time points post amputation of WT and *mfap4*^*∆/∆*^ fins. White dotted lines outline the amputation site. (**c**) Graphical representation of mean neutrophil numbers in the imaged area near the amputation site [as shown in (**b**)] of ten biological replicates per genotype for various time points post amputation. Quantification of neutrophil numbers reveals significantly more neutrophils are recruited to the amputation site in *mfap4*^∆/∆^ mutants as compared to WT larvae at most analyzed time points (0 hours post amputation (hpa), *P* = 0.0021; 0.5 hpa, *P* = 0.0002; 1 hpa, *P* < 0.0001; 2 hpa, *P* = 0.1051; 3 hpa, *P* < 0.0001; 6 hpa; *P* = 0.0002; assessed by Mann–Whitney-U test). (**d**) Quantification of neutrophil numbers in the larval tail near the amputation site at the time points shown in (**c**) relative to uncut tails. The recruitment of neutrophils is not accelerated in *mfap4*^∆/∆^ mutants. Error bars in (**b**) and (**c**) represent the standard error of the mean (s.e.m.) of ten biological replicates. Graphs in panels (**c**) and (**d**) were generated with Microsoft Excel (for Mac 2011, version 14.7.7). This figure was created with Inkscape software version 0.92 (available at https://www.inkscape.org/).

Previous studies in mice uncovered important interactions between macrophages and neutrophils during wound healing^34,35^. Neutrophils are the first leukocytes recruited to the site of injury where they migrate from blood vessels to promote inflammation and to destroy foreign material by phagocytosis^36^. We therefore assessed neutrophil count in *mfap4*^∆/*∆*^ mutant larvae after tail fin amputation using whole-mount immunofluorescence for the neutrophil-specific marker Mpx (Myeloid-specific peroxidase). Neutrophil numbers were four-fold higher in *mfap4*^∆/*∆*^ mutant larvae as compared to WT larvae prior to amputation (Fig. 4b,c, 0 hpa). Upon tail fin primordium amputation, a significantly larger number of neutrophils was recruited to the site of injury in *mfap4*^∆/*∆*^ larvae as compared to WT (Fig. 4b,c, later time points). However, the recruitment of neutrophils to the amputation site follows similar dynamics in WT and mutant larvae (Fig. 4d). In conclusion, *mfap4*^*∆/∆*^ mutant zebrafish have significantly reduced numbers of macrophages with a concomitant increase in the number of neutrophils.

### Loss of *mfap4* alters haematopoiesis in zebrafish

**Figure 5:**
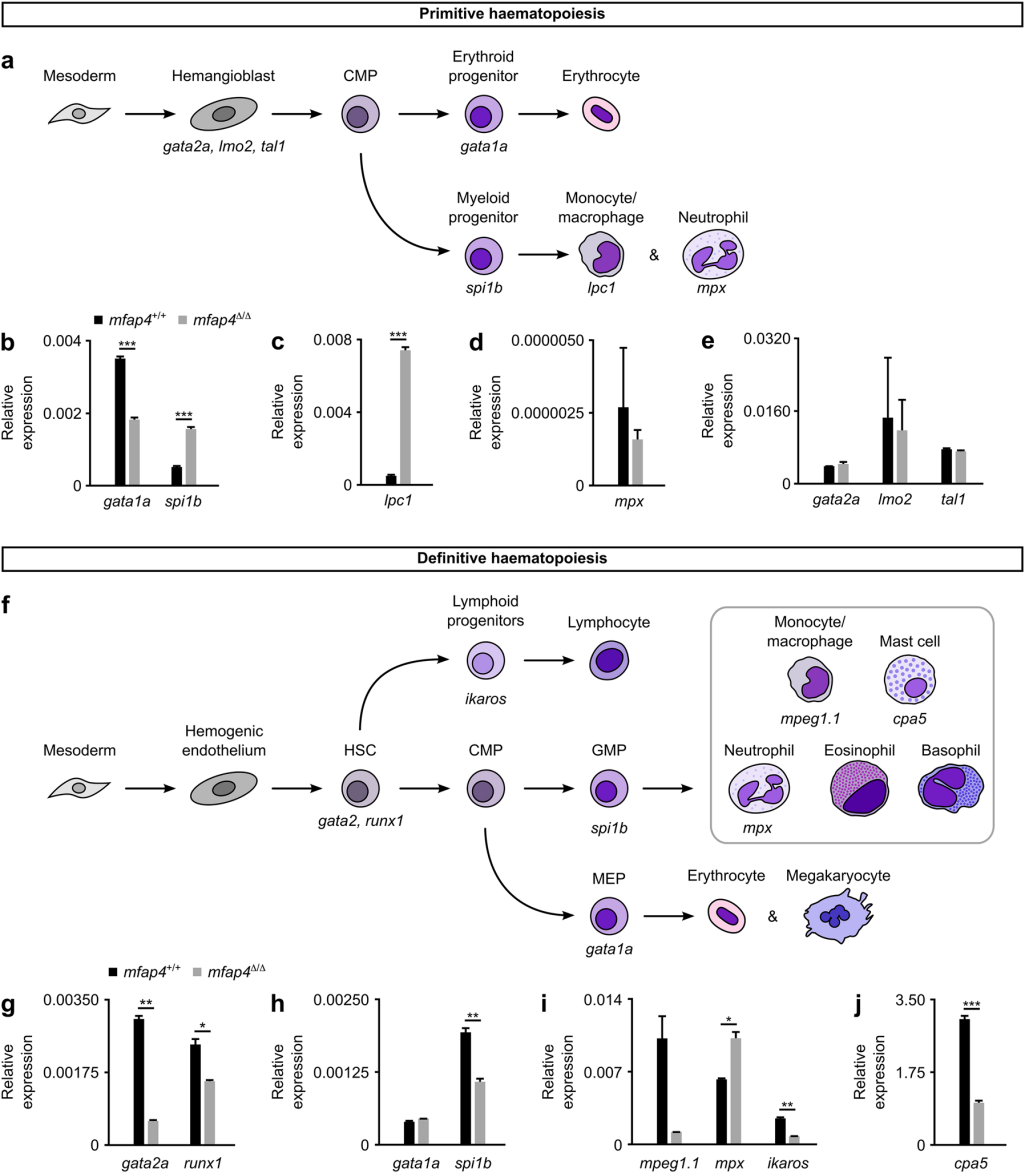
Loss of Mfap4 alters myeloid haematopoiesis in zebrafish. (**a**) Schematic of the primitive wave of zebrafish haematopoiesis (reviewed in^47,48^). (**b**–**e**) Expression of markers specific to the primitive wave in 10–14 somite stage WT (*mfap4*^+/+^) or mutant (*mfap4*^*∆/∆*^) embryos relative to the average expression of housekeeping genes (*actb1* and *eukaryotic translation elongation factor 1 alpha 1, like 1* (*eef1a1*)). In mutants, *gata1a* expression is significantly reduced (*P* = 0.000157; Student’s *t*-test), while expression of *spi1b* and *lpc1* (*P* = 0.000147 and *P* = 0.000515, respectively; Student’s *t*-test) are increased. Expression of *mpx*, *gata2a* (*P* = 0.7 for both; Mann–Whitney-U test), *lmo2* and *tal1* (*P* = 0.855612 and *P* = 0.254594, respectively; Student’s *t*-test) are not significantly affected by loss of Mfap4. (**f**) Schematic of the definitive wave of zebrafish haematopoiesis (reviewed in^47,48^). (**g**–**j**) Expression of markers specific to the definitive wave in 7-day-old WT or mutant larvae, relative to the average expression of housekeeping genes (*actb1* and *eef1a1*). At 7 dpf, expression of *gata2a*, *runx1* and *spi1b* (*P* = 0.001146, *P* = 0.02048, and *P* = 0.002625, respectively; Student’s *t*-test) is significantly reduced. Expression of *gata1a* at 7 dpf is unaffected by Mfap4 loss (*P* = 0.136085; Student’s *t*-test). Expression of *mpeg1.1* is considerably, yet not significantly, reduced in mutants (*P* = 0.1; Mann–Whitney-U test). Expression of *mpx* is significantly increased in mutants (*P* = 0.022413; *t*-test), while *ikaros* and *cpa5* expression is significantly reduced (*P* = 0.001643 and *P* = 0.000204, respectively; Student’s *t*-test). Error bars represent the standard error of the mean (s.e.m.) of technical triplicates of twenty pooled embryos/larvae per genotype per time point. Abbreviations: HSC, hematopoietic stem cell; CMP, common myeloid progenitor; MEP, megakaryocyte/erythroid progenitor; GMP, granulocyte/macrophage progenitor. Graphs in panels (**b**–**e**) and (**g**–**j**) were generated with Microsoft Excel (for Mac 2011, version 14.7.7). This figure was created with Inkscape software version 0.92 (available at https://www.inkscape.org/).

The altered macrophage and neutrophil counts in *mfap4*^*∆/∆*^ mutant fish suggest that *mfap4* plays a role during haematopoiesis. In zebrafish, haematopoiesis consists of two distinct phases—the primitive and definitive waves^30^. During early somitogenesis, ventral mesoderm differentiates into hemangioblasts^31^. The primitive wave begins around the 3 ss when hemangioblasts undergo differentiation and specification events to generate common myeloid progenitors (CMPs, Fig. 5a), which give rise to primitive erythrocytes and granulocytes/monocytes^32^. Primitive erythrocytes promote tissue oxygenation and further embryonic development, while primitive macrophages protect the embryo against invading microorganisms in the presence of injury^30^. Commitment to either the erythroid or the myeloid lineage is regulated by the transcription factors Gata1a (GATA binding protein 1a) and Spi1b/Pu.1, respectively^37^. Previous reports indicated that Gata1a and Spi1b/Pu.1 physically interact, resulting in reciprocal inhibition of their respective transcriptional targets^30^. During the primitive wave at the 10–14 ss, *gata1a* is highly enriched in erythroid progenitors, and consequently *spi1b/pu.1* expression in myeloid progenitors is repressed (Fig. 5a). However, in *mfap4*^*∆/∆*^ mutants, the expression of *gata1a* is downregulated, while *spi1b/pu.1* is upregulated three-fold (Fig. 5b). Consistent with this observation, the expression of the early macrophage commitment marker *l-plastin/lpc1* (*lymphocyte cytosolic protein 1*) showed a 14-fold increase in *mfap4*^*∆/∆*^ mutants (Fig. 5c)^38^. However, there is no statistically significant change in the expression of the neutrophil-specific marker *mpx* (Fig. 5d). Next, we analyzed the expression of a suite of hemangioblast marker genes including *gata2a*, *lmo2* (*LIM domain only 2*) and *tal1/scl* (*stem cell leukemia T-cell acute lymphocytic leukemia 1*), all of which were unaltered in *mfap4*^*∆/∆*^ mutant larvae at the 10 to 14 ss when compared to WT (Fig. 5e)^39^. Taken together, these findings suggest that Mfap4 plays a role in development of the myeloid lineage during the primitive wave of haematopoiesis.

The transition from primitive to definitive haematopoiesis begins shortly after the start of blood circulation at 24 hpf^33^. The definitive wave generates hematopoietic stem cells (HSCs) that reside in the kidney marrow where they undergo self-renewal and differentiate into erythroid, myeloid and lymphoid lineages throughout the life of the fish (Fig. 5f)^32^. Runx1 (Runt-related transcription factor 1) is essential for the specification of HSCs from the hemogenic endothelium^40^, and has been reported to initiate HSC commitment through Notch signaling in a Gata2a-dependent manner^41^. At 7 dpf, we observed a significant reduction in the expression of *runx1* and *gata2a* in *mfap4*^*∆/∆*^ mutant larvae (Fig. 5g). In addition to its role in HSC differentiation, Runx1 forms a negative feedback loop with Spi1b/Pu.1, controlling commitment to the macrophage versus neutrophil lineages^42^. High levels of Spi1b/Pu.1 promote macrophage development at the expense of neutrophil development^42^. Unlike its expression during the primitive wave, *spi1b/pu.1* expression is reduced during the definitive wave in *mfap4*^*∆/∆*^ mutant larvae (Fig. 5h). Furthermore, Gata2a and Spi1b/Pu.1 are essential for early mast cell lineage commitment^43^. Consistent with this, the expression of the mast cell progenitor marker *cpa5* (*carboxypeptidase A5*) is reduced in *mfap4*^*∆/∆*^ mutant larvae (Fig. 5j). Lymphoid progenitors that differentiate from HSC are labeled by *ikaros* (*IKAROS family zinc finger 1*), whose expression levels are also reduced in *mfap4*^*∆/∆*^ mutant larvae (Fig. 5i)^44^. As for myeloid cells, the neutrophil marker *mpx* has significantly higher expression in *mfap4*^*∆/∆*^ as compared to WT. *mpeg1.1* mRNA levels trended lower in mutant larvae (20 sampled) but did not however achieve statistical significance (Fig. 5i). When taken together, our data suggest that the loss of *mfap4* alters the balance between myeloid and lymphoid lineages during definitive haematopoiesis. Although *gata1a* expression is reduced during the primitive wave (Fig. 5b), *gata1a* expression is not significantly altered in *mfap4*^∆/∆^ mutant larvae (Fig. 5h). As the number of early erythroid cells, polychromatophils, is not altered significantly in adult *mfap4*^∆/∆^ mutants (Supplementary Fig. S4), the effect of *mfap4* loss on erythropoiesis remains unclear.

## Discussion

Apart from their crucial role in the innate immune response, macrophages and neutrophilic granulocytes are involved in wound healing and tissue regeneration^28,45^. For instance, previous studies have demonstrated that depletion of macrophages during different phases of wound healing impairs wound closure and tissue regeneration^26^. In this study, we show that in zebrafish loss of *mfap4,* which is conspicuously expressed in macrophages, results in an average of 1.5 times reduction of the total number of macrophages (Fig. 3) and a concomitant increase in the number of neutrophils (Fig. 4). Using a tail fin amputation model to investigate tissue repair, we further demonstrate that the recruitment of these two cell types to the injury site in *mfap4*^*∆/∆*^ mutant fish is unaffected and amputated fins regenerate normally (Fig. 3). Similar to our *mfap4*^*∆/∆*^ mutant zebrafish, other in vivo models in which macrophage differentiation is affected, such as *interferon regulatory factor 8* (*irf8*) mutant fish, are viable and do not exhibit an overt phenotype^46^.

Our analysis of the expression of key marker genes (*gata1a, gata2a*, *runx1* and *spi1b/pu.1*) suggests that Mfap4 plays a regulatory role during zebrafish haematopoiesis^47,48^. Spi1b/Pu.1 and Gata1a physically interact and function antagonistically to specify erythroid and myeloid cell fates^30^. Upon binding GATA1, human SPI1/PU.1 was previously shown to displace the transcriptional activator CREB-binding protein (CBP) to repress GATA1 target genes^49^. Our results show that during the primitive wave, *spi1b/pu.1* expression increases in *mfap4*^*∆*/*∆*^ mutants, which we predict inhibits the expression of Gata1a target genes and tips the balance toward the macrophage lineage, as evidenced by increased levels of *l-plastin/lpc1* mRNA (Fig. 5b,c). Jin et al.^42^ reported that high levels of Spi1b/Pu.1 promote macrophage lineage fate, while low levels of Spi1b/Pu.1 support neutrophil production^42^. Consistent with these findings, we observe that reduced levels of *spi1b/pu.1* during definitive haematopoiesis are associated with an upregulation of neutrophil but downregulation of macrophage marker genes (Fig. 5g–i).

Gata2a and Runx1 play significant roles in the specification and differentiation of HSC upstream of the CMP (Fig. 5f)^41^. During definitive haematopoiesis, *gata2a* and *runx1* expression was reduced in *mfap4*^*∆*/∆^ mutant larvae (Fig. 5g). Due to its sequence similarity with angiopoietin (Angptl), previous in vitro studies incorporated purified MFAP4 as a growth factor for the ex vivo expansion of mouse HSCs^50^. Like Mfap4, Angptl contains a C-terminal fibrinogen-like domain, and zebrafish Angptl2 interacts with Notch to in turn activate target genes such as *gata2a*^41,51^. These findings raise the intriguing possibility that these two extracellular proteins function similarly in vivo, playing a role in the development of definitive HSCs through the regulation of *gata2a* and *runx1*. In support of this, the Mfap4 relative Mfap2 induces the shedding of the Notch1 extracellular domain and activates Notch signaling in vitro^52^.

Alternatively, given the physical association between the MFAP family of proteins and fibrillins, who have been extensively described for their regulation of the bio-availability of assorted growth factors (GFs) (reviewed in^53–55^), it is possible that Mfap4 is similarly involved in the extracellular regulation of growth factor signaling. MFAP2 has indeed been shown to bind to active, but not latent, forms of members of the Transforming Growth Factor β (TGFβ) superfamily of signaling cytokines in vitro, including TGFβ1 and Bone Morphogenetic Protein 7 (BMP7)^56,57^. A connection between Mfap4 and the TGFβ signaling axis is intriguing given that we first identified *MFAP4* as a potential Activin/Nodal target gene in differentiating hESC^15,16^. It is well established that in the early mouse embryo, Nodal signaling gradients are shaped not only by the auto-regulation of *Nodal* transcription itself but also by the direct activation of genes encoding secreted Nodal antagonists and binding proteins, such as Lefty1/2 and Cerberus^58^. Perhaps zebrafish Mfap4 also serves to refine TGFβ signaling by ligand sequestration or concentration. Additional molecular studies are nevertheless needed to elucidate how Mfap4 in the ECM impacts the regulation of hematopoietic genes in the nucleus.

The human MFAP4 protein localizes principally to elastic fibers located in adult blood vessels^11^. This is in striking contrast to zebrafish *mfap4* where expression resolves to macrophages by 24 hpf. To date, there are no reports describing the spatiotemporal distribution of *Mfap4* transcripts by WISH in the developing mouse embryo or enrichment of expression in mammalian macrophages. Thus, there appear to be prominent species-specific differences between the piscine and mammalian Mfap4 orthologs. These differences are further underscored by the lack of the RGD sequence in zebrafish Mfap4. Nevertheless, both zebrafish *mfap4* and mouse *Mfap4* loss-of-function mutations result in homozygous viability^14,59^. Interestingly, zebrafish *mfap2*, like *mfap4*, is expressed in the somites during segmentation^60^, which raises the possibility that the absence of a more pronounced *mfap4* null mutant phenotype is due to compensation by *mfap2* during early development. By 1 dpf, however, *mfap2* transcripts label the caudal somites and the trunk mesenchyme as well as the connective tissue surrounding the dorsal aorta and posterior cardinal vein^60^, whereas *mfap4* is expressed specifically in macrophages. Given the expression in these distinct tissue compartments, it is difficult to envision the rescue of *mfap4*^*∆*/∆^ mutants by Mfap2 at later stages of development. It is important to note however that in the mouse *Mfap2* is widely expressed in mesodermally derived connective tissue^61^ and that inactivation of *Mfap2* results in monocytopenia, with fewer circulating monocytes^55^. The molecular mechanism underlying this phenotype is as yet unknown. Lastly, the expression of *mfap1*, *mfap3l* and *mfap5* remains poorly described (https://www.zfin.org) and invites additional experiments to understand the interrelationships of the remaining zebrafish Mfap family members.

## Methods

### Zebrafish breeding and maintenance

Zebrafish were bred and reared at the Institute of Molecular and Cellular Biology (IMCB) zebrafish facility in compliance with approved IACUC #161172 (“Breeding and maintenance of fish stocks for research use by IMB investigators and providing microinjection capabilities”) administered by the Agency for Science, Technology and Research (A*STAR) Biological Resource Centre (BRC) (https://www.a-star.edu.sg/brc/a-star-iacuc). Larvae were kept until 14 days post fertilization (dpf) in E3 medium (0.1 mM NaCl, 3.4 µM HCl, 6.6 µM CaCl_2_ and 6.6 µM MgSO_4_, calibrated to pH 7.4) at 28.5 °C before transfer into a closed water system. All experimental protocols were carried out in accordance with relevant guidelines and regulations.

### Genotype analysis

Dermal scales were isolated from adult zebrafish and lyzed overnight at 56 °C in lysis buffer (100 mM Tris–HCl pH 7.5, 50 mM EDTA pH 8.0, 0.5% SDS and 0.5 mg/mL Proteinase K). Genomic DNA was extracted using phenol:choloform:isoamyl alcohol (Merck-Calbiochem, 516,726) and dissolved in nuclease-free water. GoTaq DNA Polymerase (Promega, M5123) was used for PCR amplification of extracted DNA. Primers used to amplify WT and *mfap4*^*∆150*^ genomic DNA (indicated in Fig. 2a) are listed in Supplementary Table S1.

### Whole-mount in situ hybridization

Whole-mount in situ hybridization (WISH) was performed as previously described^62^. In brief, the *mfap4* antisense riboprobe sequence was amplified from WT 1 dpf larval cDNA and subsequently in vitro transcribed using T7 polymerase. Primers used in Figs. 1 and 2 are listed in Supplementary Table S1. Images were taken with a Leica M80 stereomicroscope with Plan 1.0X lens and DFC425 5 megapixel camera, operated with Leica Application Suite (LAS) software version 4.9.

### RNA extraction and quantitative PCR

At desired embryonic stages, 20 dechorionated embryos were pooled per time point, homogenized in TRIzol (Ambion, 15,596,018) using a 23G needle, chloroform/isopropanol precipitated and resuspended in RNase-free water. Purified RNA samples were DNase I (New England Biolabs, M0303S) treated prior to cDNA synthesis. The High-Capacity cDNA Reverse Transcription Kit (Applied Biosystems, 4,368,814) was used to synthesize 2 µg of RNA into cDNA. Quantitative PCR (qPCR) reactions were carried out using SYBR Select Master Mix (Applied Biosystems, 4,472,919). A two-sided Student’s *t*-test was used to determine statistical significance. Primers used are listed in Supplementary Table S1.

### Tissue dissociation and flow cytometry

The *Tg(mpeg1:EGFP)* fluorescent macrophage reporter was previously described (https://zfin.org/ZDB-TGCONSTRCT-120117-1)^29^ and was crossed into the *mfap4*^∆/∆^ background. Subsequent generations were identified by *Tg(mpeg1:EGFP)* fluorescence and genotyped for *mfap4*^*∆*^ as described above. Adult zebrafish were anaesthetized in Tricaine, and tail fins carefully cut within 3–5 mm from the fin cleft under a dissecting microscope. Amputated adult tail fins or entire larvae were dissociated in Hank’s solution containing 100 mg/mL collagenase type IV and 30 mg/mL protease type XIV for 1 h at 30 °C with agitation (600 rpm). Samples were gently triturated with an 18G needle every 15 min. Cells were further dissociated with 0.05% trypsin–EDTA (Gibco, 25,200,056) for 10 min under the same conditions. Prior to flow cytometry, dissociated cells were rinsed and quenched with 2% fetal bovine serum (FBS) and 2 mM EDTA in 1X PBS. For both adult tail fins and entire larvae, the number of events was set at 100,000. Data analysis was performed with FlowJo V10 software (FlowJo LLC).

### Whole-mount immunofluorescence

Whole-mount immunofluorescence was performed with an anti-Mpx antibody (GeneTex, GTX128379) at a 1:250 dilution and a goat anti-rabbit Alexa488-conjugated secondary antibody (Thermo Fisher Scientific, A21212) at a 1:500 dilution. Fin images were captured at 10X with an Olympus IX81 confocal microscope with FV1000 scanner head. GFP-positive cells were counted with Fiji software (ImageJ version 2.0.0-rc-15/1.49 k). ImageJ was used for quantification.

### Statistics

Mean values of technical triplicates of RT-PCR ΔΔ Ct values, and of biological replicates of flow cytometry counts and fluorescence microscopy neutrophil count were assessed per genotype for normal distribution by Shapiro–Wilk test and subsequently assessed for statistically significant differences between genotypes (per time point, where applicable) by either two-sampled, non-pooled, two-tailed Student’s *t*-test following t-distribution, or two-tailed Mann–Whitney-U test (Statistics Study version 4.31; Statext LLC, Carlstadt, New Jersey, USA). For all tests, a *P*-value < 0.05 was used as cut-off point to reject H_0_.

## Supplementary information

Supplementary Information.

## Data Availability

The datasets generated and analyzed during the current study are available from the corresponding author on request.

## References

[1] Elsea SH, Girirajan S (2008). Smith-Magenis syndrome. Eur. J. Hum. Genet..

[2] Hitz MP (2012). Rare copy number variants contribute to congenital left-sided heart disease. PLoS Genet..

[3] Zhao H (2019). High expression levels of AGGF1 and MFAP4 predict primary platinum-based chemoresistance and are associated with adverse prognosis in patients with serous ovarian cancer. J. Cancer.

[4] Chang PY (2017). An epigenetic signature of adhesion molecules predicts poor prognosis of ovarian cancer patients. Oncotarget.

[5] Yang J (2019). Integrated analysis of microfibrillar-associated proteins reveals MFAP4 as a novel biomarker in human cancers. Epigenomics.

[6] Davalieva K (2015). Proteomics analysis of malignant and benign prostate tissue by 2D DIGE/MS reveals new insights into proteins involved in prostate cancer. Prostate.

[7] Mölleken C (2016). MFAP4: a candidate biomarker for hepatic and pulmonary fibrosis?. Sarcoidosis Vasc. Diffuse Lung. Dis..

[8] Johansson SL (2014). Microfibrillar-associated protein 4: a potential biomarker of chronic obstructive pulmonary disease. Respir. Med..

[9] Pilecki B (2016). Characterization of microfibrillar-associated protein 4 (MFAP4) as a tropoelastin- and fibrillin-binding protein involved in elastic fiber formation. J. Biol. Chem..

[10] Niu D (2011). Microfibrillar-associated protein 4 (MFAP4) genes in catfish play a novel role in innate immune responses. Dev. Comp. Immunol..

[11] Wulf-Johansson H (2013). Localization of microfibrillar-associated protein 4 (MFAP4) in human tissues: clinical evaluation of serum MFAP4 and its association with various cardiovascular conditions. PLoS ONE.

[12] Fagerberg L (2014). Analysis of the human tissue-specific expression by genome-wide integration of transcriptomics and antibody-based proteomics. Mol. Cell Proteomics.

[13] Toyoshima T (1999). Ultrastructural distribution of 36-kD microfibril-associated glycoprotein (MAGP-36) in human and bovine tissues. J. Histochem. Cytochem..

[14] Schlosser A (2016). MFAP4 promotes vascular smooth muscle migration, proliferation and accelerates neointima formation. Arterioscler Thromb. Vasc. Biol..

[15] Teo AK (2012). Activin and BMP4 synergistically promote formation of definitive endoderm in human embryonic stem cells. Stem Cells.

[16] Brown S (2011). Activin/Nodal signaling controls divergent transcriptional networks in human embryonic stem cells and in endoderm progenitors. Stem Cells.

[17] Teo AK (2011). Pluripotency factors regulate definitive endoderm specification through eomesodermin. Genes Dev.

[18] Walton EM, Cronan MR, Beerman RW, Tobin DM (2015). The macrophage-specific promoter mfap4 allows live, long-term analysis of macrophage behavior during mycobacterial infection in Zebrafish. PLoS ONE.

[19] Zakrzewska A (2010). Macrophage-specific gene functions in Spi1-directed innate immunity. Blood.

[20] Oehlers SH (2015). Interception of host angiogenic signalling limits mycobacterial growth. Nature.

[21] Taylor JS, Braasch I, Frickey T, Meyer A, Van de Peer Y (2003). Genome duplication, a trait shared by 22000 species of ray-finned fish. Genome Res..

[22] Zhao Z (1995). The gene for a human microfibril-associated glycoprotein is commonly deleted in Smith-Magenis syndrome patients. Hum. Mol. Genet..

[23] Herbomel P, Thisse B, Thisse C (1999). Ontogeny and behaviour of early macrophages in the zebrafish embryo. Development.

[24] Popp MW, Maquat LE (2016). Leveraging rules of nonsense-mediated mRNA decay for genome engineering and personalized medicine. Cell.

[25] El-Brolosy MA, Stainier DYR (2017). Genetic compensation: a phenomenon in search of mechanisms. PLoS Genet..

[26] Koh TJ, DiPietro LA (2011). Inflammation and wound healing: the role of the macrophage. Expert Rev. Mol. Med..

[27] Perlin JR, Robertson AL, Zon LI (2017). Efforts to enhance blood stem cell engraftment: recent insights from zebrafish hematopoiesis. J. Exp. Med..

[28] Petrie TA, Strand NS, Yang CT, Rabinowitz JS, Moon RT (2014). Macrophages modulate adult zebrafish tail fin regeneration. Development.

[29] Ellett F, Pase L, Hayman JW, Andrianopoulos A, Lieschke GJ (2011). Mpeg1 promoter transgenes direct macrophage-lineage expression in zebrafish. Blood.

[30] Gore AV, Pillay LM, Venero Galanternik M, Weinstein BM (2018). The zebrafish: a fintastic model for hematopoietic development and disease. Wiley Interdiscip. Rev. Dev. Biol..

[31] Xiong JW (2008). Molecular and developmental biology of the hemangioblast. Dev. Dyn..

[32] Sood R, Liu P (2012). Novel insights into the genetic controls of primitive and definitive hematopoiesis from zebrafish models. Adv. Hematol..

[33] Li J, Li K, Dong X, Liang D, Zhao Q (2014). Ncor1 and Ncor2 play essential but distinct roles in zebrafish primitive myelopoiesis. Dev. Dyn..

[34] Daley JM (2005). Modulation of macrophage phenotype by soluble product(s) released from neutrophils. J. Immunol..

[35] Peters T (2005). Wound-healing defect of CD18(–/–) mice due to a decrease in TGF-beta1 and myofibroblast differentiation. EMBO J..

[36] Martin P, Feng Y (2009). Inflammation: wound healing in zebrafish. Nature.

[37] Cantor AB, Orkin SH (2002). Transcriptional regulation of erythropoiesis: an affair involving multiple partners. Oncogene.

[38] Lieschke GJ, Oates AC, Crowhurst MO, Ward AC, Layton JE (2001). Morphologic and functional characterization of granulocytes and macrophages in embryonic and adult zebrafish. Blood.

[39] Gering M, Yamada Y, Rabbitts TH, Patient RK (2003). Lmo2 and Scl/Tal1 convert non-axial mesoderm into haemangioblasts which differentiate into endothelial cells in the absence of Gata1. Development.

[40] Kim AD (2014). Discrete Notch signaling requirements in the specification of hematopoietic stem cells. EMBO J.

[41] Butko E (2015). Gata2b is a restricted early regulator of hemogenic endothelium in the zebrafish embryo. Development.

[42] Jin H (2012). Runx1 regulates embryonic myeloid fate choice in zebrafish through a negative feedback loop inhibiting Pu.1 expression. Blood.

[43] Dobson JT (2008). Carboxypeptidase A5 identifies a novel mast cell lineage in the zebrafish providing new insight into mast cell fate determination. Blood.

[44] Willett CE, Kawasaki H, Amemiya CT, Lin S, Steiner LA (2001). Ikaros expression as a marker for lymphoid progenitors during zebrafish development. Dev. Dyn..

[45] Crowhurst MO, Layton JE, Lieschke GJ (2002). Developmental biology of zebrafish myeloid cells. Int. J. Dev. Biol..

[46] Shiau CE, Kaufman Z, Meireles AM, Talbot WS (2015). Differential requirement for irf8 in formation of embryonic and adult macrophages in zebrafish. PLoS ONE.

[47] Orkin SH, Zon LI (2008). Hematopoiesis: an evolving paradigm for stem cell biology. Cell.

[48] Jing L, Zon LI (2011). Zebrafish as a model for normal and malignant hematopoiesis. Dis. Model Mech..

[49] Graf T, Enver T (2009). Forcing cells to change lineages. Nature.

[50] Zhang CC (2006). Angiopoietin-like proteins stimulate ex vivo expansion of hematopoietic stem cells. Nat. Med..

[51] Lin MI (2015). Angiopoietin-like proteins stimulate HSPC development through interaction with notch receptor signaling. Elife.

[52] Miyamoto A, Lau R, Hein PW, Shipley JM, Weinmaster G (2006). Microfibrillar proteins MAGP-1 and MAGP-2 induce Notch1 extracellular domain dissociation and receptor activation. J. Biol. Chem..

[53] Thomson J (2018). Fibrillin microfibrils and elastic fibre proteins: functional interactions and extracellular regulation of growth factors. Semin. Cell Dev. Biol..

[54] Zeyer KA, Reinhardt DP (2015). Fibrillin-containing microfibrils are key signal relay stations for cell function. J. Cell Commun. Signal.

[55] Mecham RP, Gibson MA (2015). The microfibril-associated glycoproteins (MAGPs) and the microfibrillar niche. Matrix Biol..

[56] Weinbaum JS (2008). Deficiency in microfibril-associated glycoprotein-1 leads to complex phenotypes in multiple organ systems. J. Biol. Chem..

[57] Craft CS (2012). Oophorectomy-induced bone loss is attenuated in MAGP1-deficient mice. J. Cell Biochem..

[58] Hill CS (2018). Spatial and temporal control of NODAL signaling. Curr. Opin. Cell Biol..

[59] Pilecki B (2015). Microfibrillar-associated protein 4 modulates airway smooth muscle cell phenotype in experimental asthma. Thorax.

[60] Chen E, Larson JD, Ekker SC (2006). Functional analysis of zebrafish microfibril-associated glycoprotein-1 (Magp1) in vivo reveals roles for microfibrils in vascular development and function. Blood.

[61] Chen Y (1993). Structure, chromosomal localization, and expression pattern of the murine Magp gene. J. Biol. Chem..

[62] Thisse C, Thisse B (2008). High-resolution in situ hybridization to whole-mount zebrafish embryos. Nat. Protoc..

[63] Dereeper A (2008). Phylogeny.fr: robust phylogenetic analysis for the non-specialist. Nucl. Acids Res..

[64] Dereeper A (2010). BLAST-EXPLORER helps you building datasets for phylogenetic analysis. BMC Evol. Biol..

