# Supplementary material for: Microfibril-associated glycoprotein 4 (Mfap4) regulates haematopoiesis in zebrafish
Source: Sci Rep. 2020 Jul 16;10:11801. doi: 10.1038/s41598-020-68792-8 (PMC7366704; doi:10.1038/s41598-020-68792-8)
Supplement: Supplementary file 1 — Supplementary Information. [file 41598_2020_68792_MOESM1_ESM.pdf]

Microfibril-associated glycoprotein 4 (Mfap4) regulates haematopoiesis in zebrafish

Sheena L. M. Ong, Ivo J. H. M. de Vos, M. Meroshini, Yogavalli Poobalan, and N. Ray Dunn

Supplementary information

Supplemental Methods

Peripheral blood smears

Age-matched, 6.5-month-old *mfap4*$^{+/+}$ and *mfap4*$^{\Delta/\Delta}$ adult zebrafish were euthanized by Tricaine overdose, and peripheral blood was smeared on a minimum of two microscope slides per fish after amputation of the tail. Air-dried blood smears were submerged in MayGrünwald stain (Sigma, MG500) for 5 min, MilliQ water for 1 min, phosphate buffer (pH 7.20) (Sigma; P3288) for 1.5 min, and Giemsa stain (ThermoFisher, 10092013; 1 in 20 dilution) for 30 min, before being rinsed twice in MilliQ water. After air-drying, coverslips were added and sealed with Cytoseal^{TM} (ThermoScientific, 8310-16). Erythrocytes were imaged by use of an Olympus BX43F microscope with 100X UPlan FL N oil lens (Olympus; NA 1.30) and DP74 color camera (Olympus) with 20.7 Mpixel resolution, operated by cellSens Standard software 2.3 (Olympus; build 18987). Cell types were scored with Fiji software.

Statistics

Mean values of biological replicates of peripheral blood smear erythroid cell differentiation were assessed per genotype for normal distribution by Shapiro-Wilk test and subsequently assessed for statistically significant differences between genotypes by twosampled, non-pooled, two-tailed Student's t-test following t-distribution (Statistics Study version 4.31; Statext LLC, Carlstadt, New Jersey, U.S.A.). For all tests, a P-value < 0.05 was used as cut-off point to reject H_0.

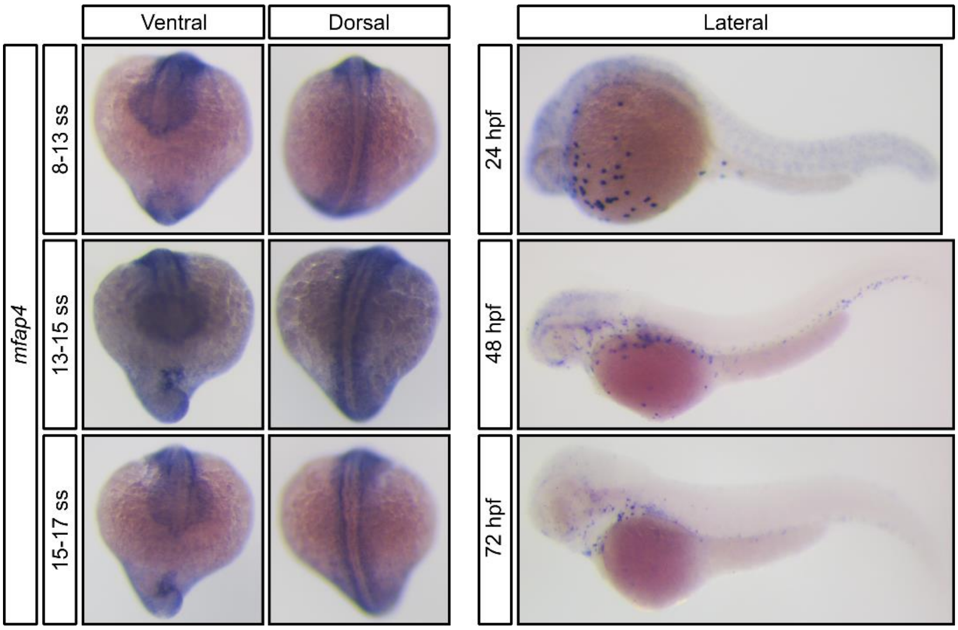

Supplementary Figure S1. Expression of *mfap4* in zebrafish embryos/ larvae. Ventral, dorsal and lateral views of *mfap4* expression in wild-type embryos/larvae, visualized by whole-mount *in situ* hybridization. Abbreviations: ss, somite stage; hpf, hours post fertilization. This figure was created with Inkscape software version 0.92 (available at inkscape.org/).

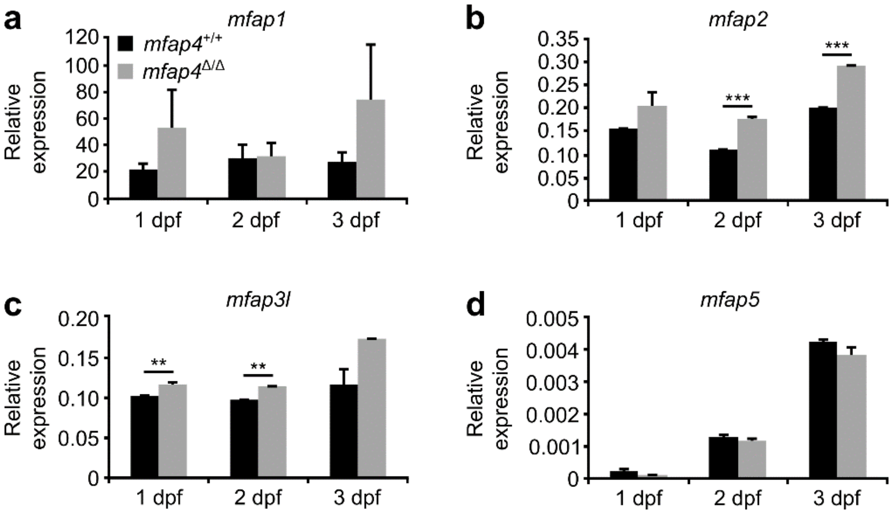

Supplementary Figure S2. Expression of *mfap* gene family members in *mfap4* mutant larvae. Quantitative RT-PCR analysis of *mfap* gene expression in 1-3 days post fertilization (dpf) wild-type (*mfap4*$^{+/+}$) and mutant (*mfap4*$^{\Delta/\Delta}$) embryos/larvae, relative to the expression of the housekeeping gene *actb1*. (a) Expression of *mfap1* is unaffected by loss of *mfap4* (1 dpf, P = 0.391059; 2 dpf, P = 0.922476; 3 dpf, P = 0.37984). (b) In *mfap4* mutants, expression of *mfap2* is increased (1 dpf, P = 0.250724; 2 dpf, P = 0.000153; 3 dpf, P = 0.000126). (c) In *mfap4* mutants, expression of *mfap3l* is increased (1 dpf, P= 0.006388; 2 dpf, P = 0.005359; 3 dpf, P = 0.238299). (d) The expression of *mfap5* is not significantly affected by loss of *mfap4* (1 dpf, P = 0.08112; 2 dpf, P = 0.238847; 3 dpf, P = 0.241142). Error bars represent the standard error of the mean (s.e.m.) of technical triplicates of 20 pooled embryos/larvae per genotype per time point. Differences in means per time point were assessed for significance by Student's t-test. Graphs were generated with Microsoft Excel (for Mac 2011, version 14.7.7). This figure created with Inkscape software 0.92 (available at inkscape.org/).

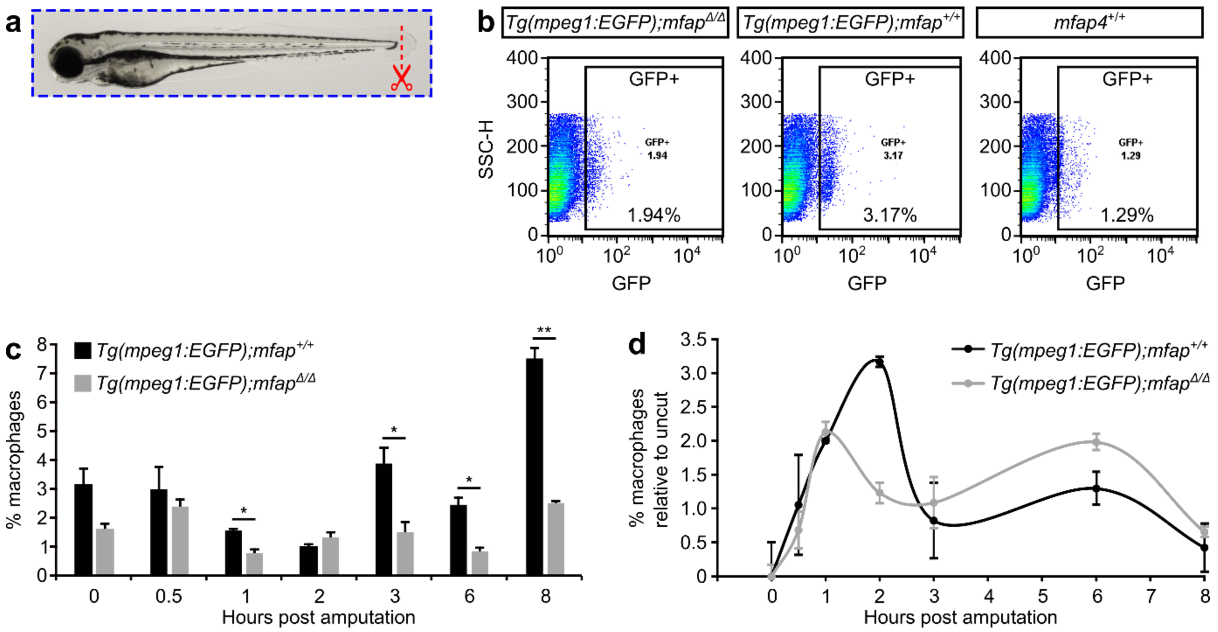

Supplementary Figure S3. *mfap4* mutant larvae have reduced number of macrophages. (a) The tail fin primordium of 3 days post fertilization (dpf) wild-type (WT, *mfap4*$^{+/+}$) and mutant (*mfap4*$^{\Delta/\Delta}$) larvae was amputated (along the red dotted line), and cells of whole larvae (blue dotted box) were dissociated and macrophage numbers quantified by FACS using the *Tg(mpeg1:EGFP)* reporter. (b) FACS analysis depicting side scatter (SSC) on the y-axis and *Tg(mpeg1:EGFP)* fluorescence on the x-axis. (c) Graphical representation of mean macrophage numbers of three biological replicates per genotype (determined as in panel (b)) for various time points post amputation. All time points were corrected for respective stages background signal. At most analysed time points, there are significantly fewer macrophages in *mfap4*$^{\Delta/\Delta}$ larvae as compared to WT larvae (0 hpa, P = 0.1077; 0.5 hpa, P = 0.521437; 1 hpa, P = 0.034479; 2 hpa, P = 0.15936; 3 hpa, P = 0.040784; 6 hpa, P = 0.01069; 8 hpa, P = 0.005174; assessed by Student's t-test). (d) Quantification of macrophage numbers in tail fins at the time points shown in (c) relative to uncut fin primordia. There is no delay in macrophage recruitment in *mfap4*$^{\Delta/\Delta}$ mutants. Error bars represent the standard error of the mean (s.e.m.) of biological replicates. Panel (b) was created with FlowJo V10 software. Graphs in panel (c) and (d) were generated with Microsoft Excel (for Mac 2011, version 14.7.7). This figure was created with Inkscape version 0.92 (available at inkscape.org/).

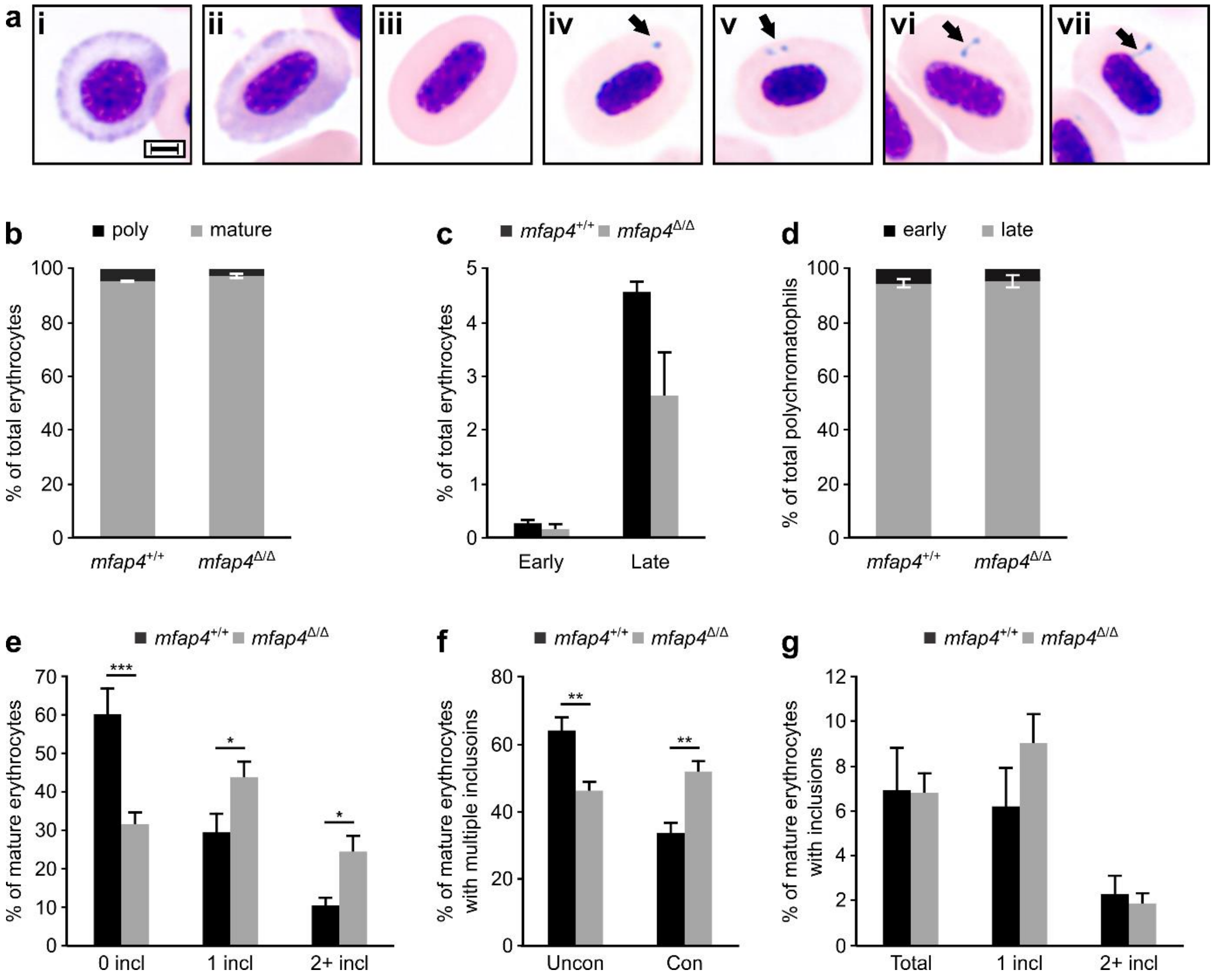

Supplementary Figure S4. Cytoplasmic inclusions are more frequently present in mature erythrocytes of adult *mfap4* mutant zebrafish, while polychromatophil morphology and relative numbers are unaffected. (a) Representative images of erythroid cells observed in May-Grünwald Giemsa stained peripheral blood smears of adult fish. The most abundant cells included early (i) and late (ii) polychromatophils, and mature erythrocytes (iii). A subset of mature erythrocytes has a single (iv) or multiple (v) cytoplasmic inclusions (arrow), which can be interconnected (vi) or connected to the nucleus as a nuclear bridge (vii). Scale bar equals 2 µm. (b-d) There are no statistically significant differences in the average percentage of total (b, P = 0.068441), early (c, P = 0.396518) or late (P = 0.066524) polychromatophils (poly) between *mfap4* mutant and wild-type (WT) fish. (d) The ratio between early and late polychromatophils does not differ significantly between mutant and WT fish (P = 0.0352108). (e) In *mfap4* mutant fish, the percentage of mature erythrocytes with a single (1 incl) or multiple (2+ incl) cytoplasmic inclusions is significantly increased (P = 0.026091 and P = 0.011398, respectively) compared to WT fish, while the subset of

Supplementary Figure S4 (cont'd). erythrocytes without inclusions is decreased (0 incl, P = 0.00093). (f) In mature erythrocytes of mutant fish with multiple cytoplasmic inclusions, the inclusions are more frequently interconnected (con) compared to WT fish (P = 0.001359). (g) The percentage of nuclear bridges in the total number of mature erythrocytes with cytoplasmic inclusions (total), a single inclusion (1 incl) or multiple inclusions (2+ incl) does not significantly differ between mutant and WT fish (P = 0.477402, P = 0.750859, and P = 0.332077, respectively). A total of 4,362 (WT) and 4,048 (mutant) erythroid blood cells were scored, obtained from five WT and six mutant adult fish, respectively. Error bars represent the standard error of the mean (s.e.m.) of biological replicates. Means were assessed for statistical differences by Student's t-test. Graphs in panel (b-g) were generated with Microsoft Excel (for Mac 2011, version 14.7.7). This figure was created with Inkscape software version 0.92 (available at inkscape.org/).

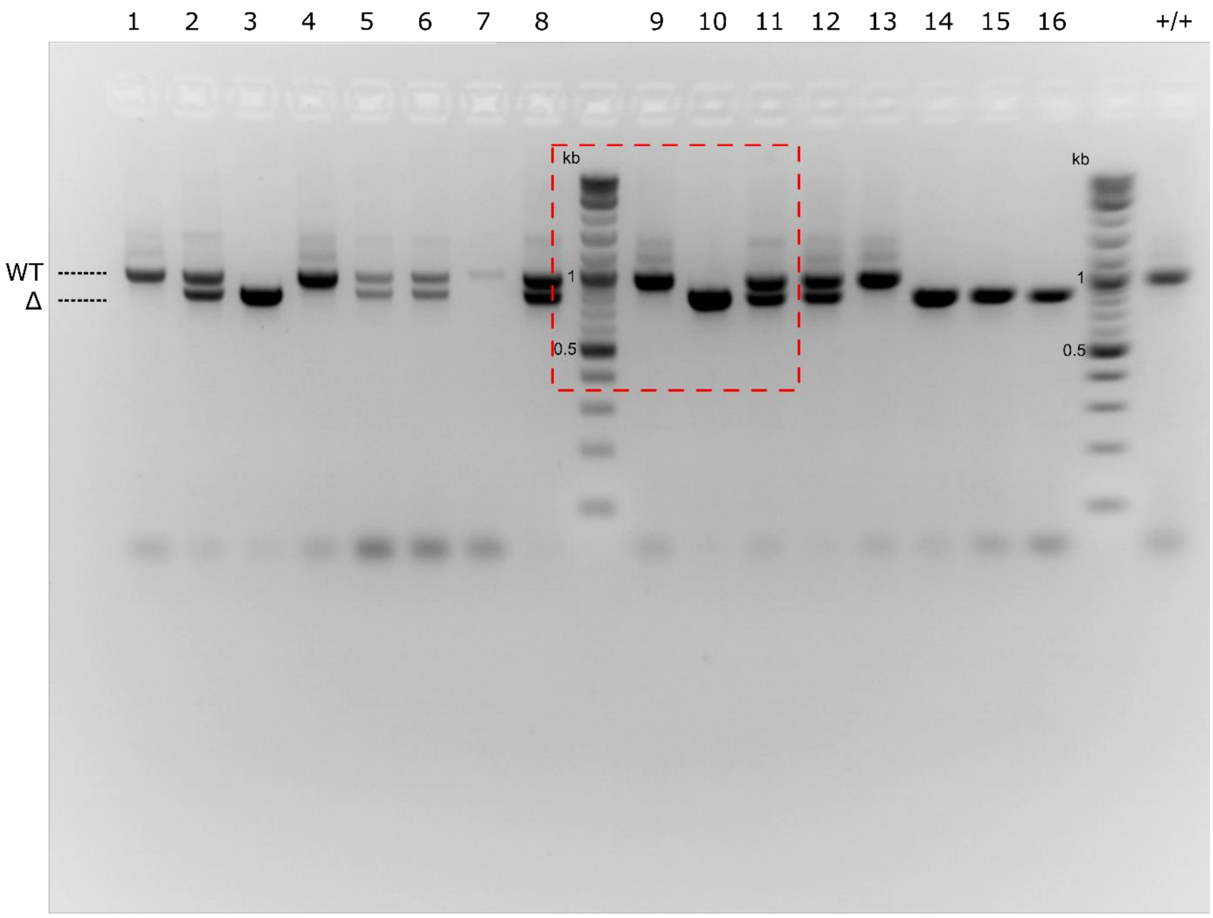

Supplementary Figure S5. Genotyping analysis of *mfap4* in zebrafish. DNA gel is loaded with DNA ladder (Lanes 1 to 16) and PCR product of individual fish visualised with ethidium bromide. The WT and mutant amplicon have a length of 946 bp and 796 bp, respectively. The red box shows the selected area for Fig. 2b, which neatly shows the genotyping results of a WT (Lane 9), homozygous (Lane 10) and heterozygous (Lane 11) fish. This figure was created with Inkscape version 0.92 (available at inkscape.org/).

Supplementary Table S1. Primer sequences for riboprobes, genotyping and Q-PCR.

Purpose	Gene	Sequence (5'-3')
Genotyping	*mfap4*	Fw – GCAGATTTGGCATAAAACGGAACC
		Rv – CCCACAGAGAAGGACGAGT
Riboprobe	*mfap4*	Fw – GTGATTCAGAGGAGGATGGAC
		Rv – ATACTGACAGTGTGAGTGCC
RT-PCR	*actb1*	Fw – CGAGCAGGAGATGGGAACC
		Rv – CAACGGAAACGCTCATTGC
	cpa5	Fw – TCGTCTACACCCACACCAAA
		Rv – TGATTTTGCCGTGAGACTTC
	eef1a1	Fw – CGGTGACAACATGCTGGAGG
		Rv – ACCAGTCTCCACACGACCCA
	gata1a	Fw – AGCGCCTGAGTCCCATGAGTG
		Rv – GGTCCCCCAGTTGAATAGAGC
	gata2a	Fw – GTAGTCAAAACGGCCACCTC
		Rv – ACAATGTGTTCCGAGGGAAA
	ikaros	Fw – TAACCTGCTCCGACACAT
		Rv – CTCCGCTGCTTGTAACTG
	lmo2	Fw – ACTACAAACTCGGCAGAAAGC
		Rv – CACGCATGGTCATTTCAAAGG
	lpc1	Fw – GCCCTTCACCATACAGGAGA
		Rv – AGCAGAGCGATCAGAGCTTC
	mfap1	Fw – GACCGAGAATCCAGAGAAGCATTG
		Rv – CTTGCTCGCCATCCATGAAGAAAG
	mfap2	Fw – GAACGCGCTGGATGTACAGAC
		Rv – TTCTGCGCAGACTGTAGAAGCAG
	mfap3l	Fw – CCATCATGTTGAGTGCATCCAC
		Rv – ATCACGTATCCGTTCACCCTCT
	mfap4	Fw – GAATACTGGCTGGGACTAGAG
		Rv – GAGTCGCCTGCTCCTCCATC
	mfap5	Fw – GCCAGACTGCAGAGAGGAAACT
		Rv – CCTGCAGATTTCAGCCATCAGGA
	mpeg1.1	Fw – CACGGGTTCAAGTCCGTAACCATC
		Rv – GCCGTAATCAAGTACGAGTT
	mpx	Fw – TGCCACAAACCTGCTCACTCAAGAC
		Rv – GAAGTGGGTTCTTCCGATTGTTGC
	spi1b	Fw – CCATTAGAGGTGTCCGATGAG
		Rv – ACCAGATGCTGTCCTTCATG
	runx1	Fw – TTGGGACGCCAAATACGAACC
		Rv – ATATCACCAAGGGCAACCACC
	tal1	Fw – CCGCTCGCCACTATTAACAG
		Rv – GTTCGTGAAAATCCGTCGC

Fw, forward primer; Rv, reverse primer.
